# Associations Between Diabetic Retinopathy and Parkinson's Disease: Results From the Catalonian Primary Care Cohort Study

**DOI:** 10.3389/fmed.2021.800973

**Published:** 2022-01-18

**Authors:** Didac Mauricio, Bogdan Vlacho, Joan Barrot de la Puente, Xavier Mundet-Tudurí, Jordi Real, Jaime Kulisevsky, Emilio Ortega, Esmeralda Castelblanco, Josep Julve, Josep Franch-Nadal

**Affiliations:** ^1^DAP-Cat Group, Unitat de Suport a la Recerca Barcelona, Fundació Institut Universitari per a la recerca a l'Atenció Primària de Salut Jordi Gol i Gurina, Barcelona, Spain; ^2^Departament of Medicine, University of Vic-Central University of Catalonia, Catalonia, Spain; ^3^Department of Endocrinology and Nutrition, Hospital de la Santa Creu i Sant Pau, Barcelona, Spain; ^4^CIBER of Diabetes and Associated Metabolic Diseases, Instituto de Salud Carlos III, Madrid, Spain; ^5^Primary Health Care Center Dr. Jordi Nadal i Fàbregas (Salt), Gerència d'Atenció Primaria, Institut Català de la Salut, Girona, Spain; ^6^Faculty of Medicine, Department of Medicine, Autonomous University of Barcelona, Barcelona, Spain; ^7^Movement Disorders Unit, Neurology Department, Hospital de la Santa Creu i Sant Pau, Barcelona, Spain; ^8^Department of Endocrinology and Nutrition, Institut d'Investigacions Biomèdiques August Pi i Suñer, Hospital Clinic, Barcelona, Spain; ^9^CIBER of Physiopathology of Obesity and Nutrition, Instituto de Salud Carlos III, Madrid, Spain; ^10^Division of Endocrinology, Metabolism and Lipid Research, Washington University School of Medicine in St. Louis, St. Louis, MO, United States; ^11^Institut de Recerca de l'Hospital de la Santa Creu i Sant Pau, Barcelona, Spain; ^12^Primary Health Care Center Raval Sud, Gerència d'Atenció Primaria, Institut Català de la Salut, Barcelona, Spain

**Keywords:** age, diabetic retinopathy, diabetes type 2, Parkinson's disease, primary care, real world data (RWD)

## Abstract

The purpose of this study was to assess the risk of occurrence of Parkinson's disease (PD) among subjects with type 2 diabetes and diabetic retinopathy (DR) in our large primary health care database from Catalonia (Spain). A retrospective cohort study with pseudo-anonymized routinely collected health data from SIDIAP was conducted from 2008 to 2016. We calculated the number of events, time to event, cumulative incidence, and incidence rates of PD for subjects with and without DR and for different stages of DR. The proportional hazards regression analysis was done to assess the probability of occurrence between DR and PD. In total, 26,453 type 2 diabetic subjects with DR were identified in the database, and 216,250 subjects without DR at inclusion. During the follow-up period, 1,748 PD events occurred. PD incidence rate and cumulative incidence were higher among subjects with DR (16.95 per 10,000 person-years and 0.83%, respectively). In the unadjusted analysis, subjects with DR were at 1.25 times higher risk (hazard ratio: 1.22, 95% confidence interval: 1.06; 1.41) of developing PD during the study period. However, we did not find any statistically significant HR for DR in any models after adjusting for different risk factors (age, sex, duration of diabetes, smoking, body mass index, glycosylated hemoglobin, comorbidities). In conclusion, in our primary health care population database, DR was not associated with an increased risk of PD after adjusting for different risk factors. In our retrospective cohort study, age, male sex, and diabetes duration were independent risk factors for developing PD.

## Introduction

Diabetes mellitus (DM) is a metabolic disorder that may result in an unfavorable impact in different organs and leading to numerous complications (1). The complications of diabetes are generally classified as macrovascular and microvascular. Large blood vessels are affected by macrovascular complications, and depending on the location, three types of complications exist: coronary artery disease, peripheral artery disease, and cerebrovascular disease. On the other hand, microvascular complications affect blood microvessels, leading to complications such as diabetic peripheral neuropathy, diabetic nephropathy, and diabetic retinopathy (DR). However, unlike classical vascular complications, DM may affect almost every organ system and damage other tissues or cell types (1). In addition to vascular tissue, damage to non-vascular tissue also happens in DR, one of the most common complications among subjects with DM. Due to the retina's particular neurovascular structure, neuro-dysfunction and neurodegeneration caused by DM is an important component of this complication. For example, it was previously reported that different neuronal damage and functional changes could occur in the retina due to poor DM control. These changes include loss of dendrites and synaptic activity, neural apoptosis, thinning of the inner retina, ganglion cell loss, reactive microglial activation, and deficits in the retina's electrophysiological activity, dark adaptation, contrast sensitivity, or color vision (2, 3). Overall, the complications of diabetes are far from vascular only, and there are a lot of other non-classical chronic complications of diabetes, including neurodegenerative complications (1).

Parkinson's disease (PD) is the second most common neurodegenerative disease. Generally, this chronic disease is more prevalent among older adults and Hispanic people (4). So far, many risk factors have been reported to be related to PD, such as pesticides, dietary factors, melanoma, traumatic brain injury, and diabetes (4–7). The role of DM as a risk factor for neurodegenerative diseases involves different pathways of cellular metabolic injury such as impaired insulin signaling and inflammatory and oxidative stress, which can lead to mitochondrial dysfunction, neuroinflammation, synaptic plasticity and other neuronal dysfunction and degeneration (8). So far, different studies have been conducted to evaluate the risk of DM on PD. In cohort studies from Finland, Denmark, UK, Taiwan, and South Korea (9–14), type 2 diabetes mellitus (T2DM) was a risk factor for PD. However, no such associations were found in cohort studies in the US (15, 16).

The results related to the association between DM and PD reported in meta-analyses have also been heterogeneous. In one meta-analysis with 11 observational studies (four cohort and seven case-control studies) on the association of diabetes as a risk factor for PD, the authors concluded that diabetes appears to be a risk factor for PD (6). Another meta-analysis with observational studies indicated that T2DM increases the risk of future PD; however, no associations were found when the authors changed the exposure definition to any type of diabetes (7). Moreover, the authors pointed out that the association between DM and PD tended to change depending on the study design since a possible effect of survivor bias among the patients with diabetes may interfere with the results (7).

Evidence suggests that DR and PD share similar pathophysiological characteristics and mechanisms (dopamine reduction, increased α-Synuclein expression, and abnormal neurotrophic factors expression) related to disrupted dopamine activity since both brain and retina express D1-like and D2-like dopamine receptors (17). The phosphorylation of α-Synuclein as a result of the dopamine in abnormal regulation in the retinal layers may be a reason for neurodegeneration in the retina and brain (18). Therefore, a close association between pathophysiological mechanisms of DR and PD may be expected.

To our knowledge, there is only one large population database study from South Korea aimed to investigate this association. The authors found that the incidence of PD was higher among DM subjects and even higher among subjects with DM and DR; however, they acknowledged that important variables related to the DM were not available for the analysis, such as duration of diabetes, and glycated hemoglobin (19). On this background, we undertook the current study to assess the risk of occurrence of PD among subjects with DR in our large primary health care database from Catalonia (Spain).

## Materials and Methods

### Study Design and Data Source

We used a retrospective cohort of subjects with T2DM attended in primary health care centers from the Catalonian Health Institute–ICS, using the pseudo-anonymized routinely collected health data from the SIDIAP (Sistema d'Informació per al desenvolupament de la Investigació en Atenció Primària) database. This database is a well-validated data source for the study of diabetes in Spain (20, 21), collecting different data related to health problems, clinical and diagnostic procedures, laboratory parameters, and information on medication prescribed and dispensed. The data were collected for the period between January 1, 2008, and December 31, 2016.

### Definition of Eligibility Criteria

We included all subjects aged 30 years or above, with a register of T2DM defined as the presence of relevant ICD-10 (International Classification of Diseases, 10th Revision) diagnostic codes and sub-codes (E11 and E14). Subjects with diagnostic codes for other types of diabetes (type 1, gestational or other) or without T2DM codes were excluded from the analysis. Those subjects with preexisting primary and secondary Parkinson's disease (ICD-10: G20 and G21) were also excluded from the study population.

### Definition of Variables

At inclusion, variables related to DR and clinical characteristics of the subjects were collected. DR was defined as the presence of diagnostic codes and sub-codes (ICD-10: E11.3, E14.3, and H36) and/or abnormal (pathologic) results for fundus photography. In those with available fundus photography data, DR was stratified in different stages using the Early Treatment Diabetic Retinopathy Study (ETDRS) classification: no apparent retinopathy (NDR), mild non-proliferative retinopathy (NPDR), moderate NPDR, severe NPDR, proliferative diabetic retinopathy (PRD), and diabetic macular edema (DME) (22). We also collected variables related to sociodemographic characteristics (age, sex) and toxic habits (tobacco use). Duration of T2DM was calculated. Due to the under-reporting in our database, dyslipidemia and hypertension were identified as a combination of diagnostic code and/or treatment for these diseases. In addition, chronic kidney disease (CKD) was defined as a combination of CKD-EPI glomerular filtration rate <60 ml/min/1.73 m^2^ and/or an albumin/creatinine ratio >30 mg/g. To identify cardiovascular disease, we used diagnostic codes alone. Moreover, concomitant medication (antihypertensive, antiplatelet, lipid-lowering, antidiabetic drugs), laboratory parameters [lipid profile, renal profile, glycated hemoglobin (HbA1c)], and clinical variables [systolic and diastolic blood pressure, body mass index (BMI)] were collected. The detailed information on the different codes used in defining the study variables is included as Table 1 code list.

**Table 1:** Code list.

**Variable**	**Definition**
**Codes used for definition of the study variables**	
Type 2 diabetes mellitus	ICD-10-CM Codes: E11.xx; E14.xx
Cardiovascular diseases	ICD-10-CM Codes:I21.xx; I22.xx; I23.xx; I25.xx; G45.xx; G46.xx; I60.xx; I61.xx I62.xx; I63.xx; I64.xx
Diabetic retinopathy	ICD-10-CM Codes: E11.3; E14.3; H36;H36.0;H36.8 and/or fundus photography: mild non-proliferative retinopathy (NPDR), moderate NPDR, severe NPDR, proliferative diabetic retinopathy (PRD), and diabetic macular edema (DME)
Dyslipidemia	ICD-10-CM Codes: E78;E78.9 and/or Lipid-lowering drugs
Hypertension	ICD-10-CM Codes: 10 and/or Antihypertensive agents
Parkinson's disease as event	ICD-10-CM Codes: G20
Parkinson's disease as exclusion criteria	ICD-10-CM Codes: G20;G21.xx
Chronic kidney disease	CKD-EPI glomerular filtration rate <60 ml/min/1.73 m^2^ and or albumin/creatinine ratio>30 mg/g
Antithrombotic agents	ATC/DDD codes: B01A
Antihypertensive agents	ATC/DDD codes: C02; C03; C07; C08; C09
Antidiabetics agents	ATC/DDD codes: A10
Lipid-Lowering agents	ATC/DDD codes: C10

*xx, sub codes*.

During follow-up, we collected data related to PD as a primary study event. PD was defined as the presence of a diagnostic code for Parkinson's disease (ICD-10: G20). The follow-up period was defined as the time between the inclusion in the study and the primary study event.

### Statistical Analysis

For the variables collected at inclusion, we used descriptive statistics. The number and frequencies for the qualitative variables were calculated, while we estimated the means and standard deviation for the quantitative variables.

At follow-up, for the primary study event (PD), we calculated the number of subjects and events for each group (presence/absence of DR and each stage of DR), time to event (time to a diagnosis of PD after a diagnosis of DR), cumulative incidence and incidence rates (person/year). To assess the probability of occurrence between DR and PD, we used proportional hazards regression analysis. The hazard ratios (HR) for the primary outcome event were calculated with corresponding 95% confidence intervals (CI), and statistical significance was established as a *p* < 0.05. Additionally, adjusted HRs were calculated using different clinically important variables as risk factors. Gradually we added different risk factors to the model, starting with age and sex in the first model, adding T2DM duration, smoking status, hypertension, dyslipidemia, and BMI in the second model, and adding CKD, CVD, and HbA1c in the third model. Moreover, to assess the effect of diabetes duration and HbA1c on the association of DR with risk of incident PD, we performed additional models with different combinations of these two variables with the other relevant variables from the first model. We also performed a sensitivity analysis with the estimates from different models and, also, stratification for diabetes duration and HbA1c. Data management and all analyses were performed using R statistical software, version 3.6.1.

### Institutional Review Board Statement

The study was conducted according to the guidelines of the Declaration of Helsinki and approved by the Institutional Review Board (or Ethics Committee) of IDIAP Jordi Gol i Gurina Foundation (protocol code P13/028 and date of approval 03/04/2013).

## Results

### Subjects Characteristics

**Figure 1:**
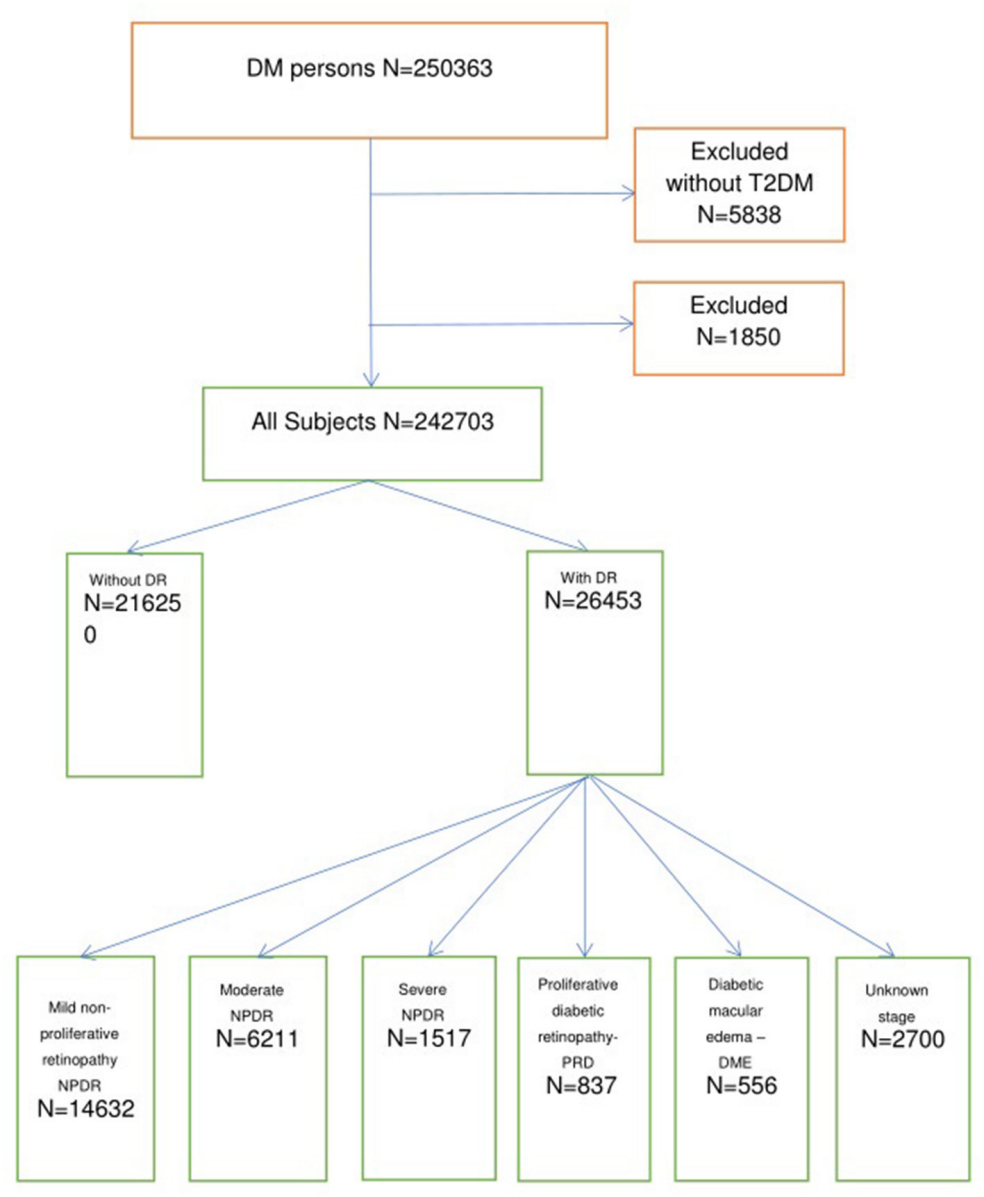
Study flowchart.

From 2008 until 2016, 250,363 subjects with T2DM were identified in the SIDIAP database. We excluded 1,850 individuals who had a previous diagnostic category of PD and 5,838 individuals without T2DM diagnostic codes. In total, 216,250 subjects did not have DR at baseline, while 26,453 had DR by diagnostic code and/or diagnosis by fundus photography. The study flowchart is presented in Figure 1.

The clinical characteristics of the cohort and different groups at inclusion are presented in Table 2. The mean age of the study subjects was 65.3 years. There were more males (57.5%), and the average duration of T2DM was 5.35 years. There were differences between the study groups regarding age, comorbidities, laboratory parameters, and concomitant drug use. Subjects with DR were, on average, 2.7 years older and with more comorbidity, especially cardiovascular diseases and CKD, compared with those without DR. We also observed higher mean values for HbA1c, lower glomerular filtration ratios, and slightly lower BMI among subjects with DR. Regarding concomitant drug use, higher percentages were also observed for all drug classes of interest in the DR group.

**Table 2:** Clinical characteristics of the subjects at study inclusion.

	**All subjects *N* = 242,703**	**Group without DR** ***N* = 216,250**	**Group with DR *N* = 26,453**	***p*-value**
Age, mean (SD), years	65.3 (11.5)	65.0 (11.5)	67.7 (11.5)	<0.001
Sex (male), *n* (%)	142,749 (57.4)	127,534 (57.5)	15,215 (56.7)	
**Smoking habit**, ***n*** **(%)**				<0.001
No smoker	140,585 (57.9)	124,530 (57.6)	16,055 (60.7)	
Ex-smoker	32,843 (13.5)	29,782 (13.8)	3,061 (11.6)	
Current smoker	69,275 (28.5)	61,938 (28.6)	7,337 (27.7)	
**Comorbidities**, ***n*** **(%)**				
Dyslipidemia	125,617 (51.8)	111,117 (51.4)	14,500 (54.8)	<0.001
Hypertension	155,115 (63.9)	136,395 (63.1)	18,720 (70.8)	<0.001
Cardiovascular diseases	30,014 (12.4)	25,424 (11.8)	4,590 (17.4)	<0.001
Chronic kidney disease	37,804 (15.6)	31,529 (14.6)	6,275 (23.7)	0.000
**Clinical variables, mean, (SD)**				
Diabetes duration, (years)	5.35 (5.39)	4.98 (5.04)	8.41 (6.92)	0.000
BMI (kg/m^2^)	30.6 (5.16)	30.6 (5.15)	30.2 (5.24)	<0.001
SBP (mmHg)	134 (14.9)	134 (14.7)	137 (16.3)	<0.001
DBP (mmHg)	76.7 (9.72)	76.9 (9.62)	75.4 (10.4)	<0.001
**Laboratory parameters, mean, (SD)**				
HbA1c (%) HbA1c (mmol/mol)	7.18 (1.52) 55.0 (16.6)	7.12 (1.48) 54.3 (16.2)	7.74 (1.72) 61.1 (18.8)	0.000
Total cholesterol (mg/dl)	195 (41.1)	195 (40.9)	188 (42.5)	<0.001
HDL cholesterol (mg/dl)	48.6 (12.8)	48.6 (12.8)	48.8 (13.2)	0.005
LDL cholesterol (mg/dl)	114 (34.3)	115 (34.2)	109 (34.9)	<0.001
Triglycerides (mg/dl)	167 (121)	168 (123)	159 (109)	<0.001
Creatinine (mg/dl)	0.91 (0.30)	0.90 (0.28)	0.96 (0.43)	<0.001
Albumin / Creatinine ratio (mg/g)	35.9 (142)	31.2 (123)	74.0 (239)	<0.001
Glomerular filtration (ml/min/1.73 m^2^)	76.7 (20.5)	77.5 (20.1)	69.4 (22.8)	<0.001
**Concomitant medications**, ***n*** **(%)**				
Antithrombotic	76,507 (31.5)	64,823 (30.0)	11,684 (44.2)	0.000
Antihypertensive	154,166 (63.5)	135,576 (62.7)	18,590 (70.3)	<0.001
Antidiabetics	181,237 (74.7)	158,665 (73.4)	22,572 (85.3)	0.000
Lipid-lowering	123,969 (51.1)	109,697 (50.7)	14,272 (54.0)	<0.001

*BMI, body mass index; SBP, systolic blood pressure; DBP, diastolic blood pressure; HbA1c, glycosylated hemoglobin; SD, standard deviation*.

### Parkinson's Disease Incidence Among the Groups

Information on the time of follow-up, events, and cumulative incidence are presented in Table 3. The average time to the event among study subjects was 4.8 years. In total, 1,748 PD events occurred, with an incidence rate of 14.25 per 10,000 person-years and a cumulative incidence of 0.72%. PD incidence and cumulative incidence were higher among subjects with DR (16.95 per 10,000 person-years and 0.82%, respectively). When the different stages of DR were compared, the highest incidence rate and cumulative incidence of PD was observed among those with moderate non-proliferative retinopathy (20.73 per 10,000 person-years and 0.99%, respectively). High incidence rate and cumulative incidence were observed among individuals with DR identified by diagnostic code but without grading of DR according to fundus photography (27.77 per 10,000 person-years and 1.33%, respectively).

**Table 3:** Parkinson's disease events among the study groups, stage of diabetic retinopathy and un-adjusted hazards ratios.

**Variable**	**N subjects**	**Person-years**	**Time free from the event (years)**	**Parkinson's disease events**	**Incidence rate per 10000-Year**	**Cumulative incidence**	**Un-adjusted HR 95% CI [LI; Ul]**
All subjects	242,703	1226699.67	4.82	1,748	14.25	0.72	–
Group without DR	216,250	1097519.23	4.86	1,529	13.93	0.71	Ref
Group with DR	26,453	129180.43	4.56	219	16.95	0.83	1.22 [1.06; 1.41]
**Stage of DR**							
No apparent diabetic retinopathy (NDR)	216,250	1097519.23	4.86	1,529	13.93	0.71	Ref
Mild non-proliferative diabetic retinopathy (NPDR)	14,632	72673.13	4.63	109	14.99	0.75	1.08 [0.89; 1.31]
Moderate (NPDR)	6,211	29900.12	4.48	62	20.74	0.99	1.50 [1.16; 1.93]
Severe (NPDR)	1,517	7669.25	4.91	3	3.91	0.20	0.28 [0.09; 0.87]
Proliferative diabetic retinopathy (PRD)	837	3736.21	4.11	5	13.38	0.60	0.98 [0.41; 2.35]
Diabetic macular edema (DME)	556	2238.91	3.56	4	17.87	0.72	1.33 [0.50; 3.54]
Unknown stage^*^	2,700	12962.82	4.65	36	27.77	1.33	2.02 [1.45; 2.81]

*DR, diabetic retinopathy; NDR, no apparent diabetic retinopathy; NPDR, non-proliferative diabetic retinopathy; PRD, proliferative diabetic retinopathy; DME, diabetic macular edema; ref, reference group; HR, hazard ratio; 95% CI, 95% confidence interval; LI, lower limit; Ul, upper limit.*

* *Subjects having diabetic retinopathy by diagnostic code but without fundus photography/stage of DR*.

### Factors Predicting Parkinson's Disease

We observed statistically significant un-adjusted HR (Unadj-HR) for the primary study event between the groups (Table 3). The subjects with DR had a 1.22 times higher risk of developing PD during the study period. Additionally, we also calculated the Unadj-HR considering the stage of DR. The highest Unadj-HR was observed among the subjects with moderate NPDR. These subjects were at a 1.50 times higher risk of developing PD compared with those without DR. Further, a higher risk (Unadj-HR: 2.02) of developing PD was observed for the group with DR identified by diagnostic code, but without grading of DR compared with the group without DR.

**Figure 2:**
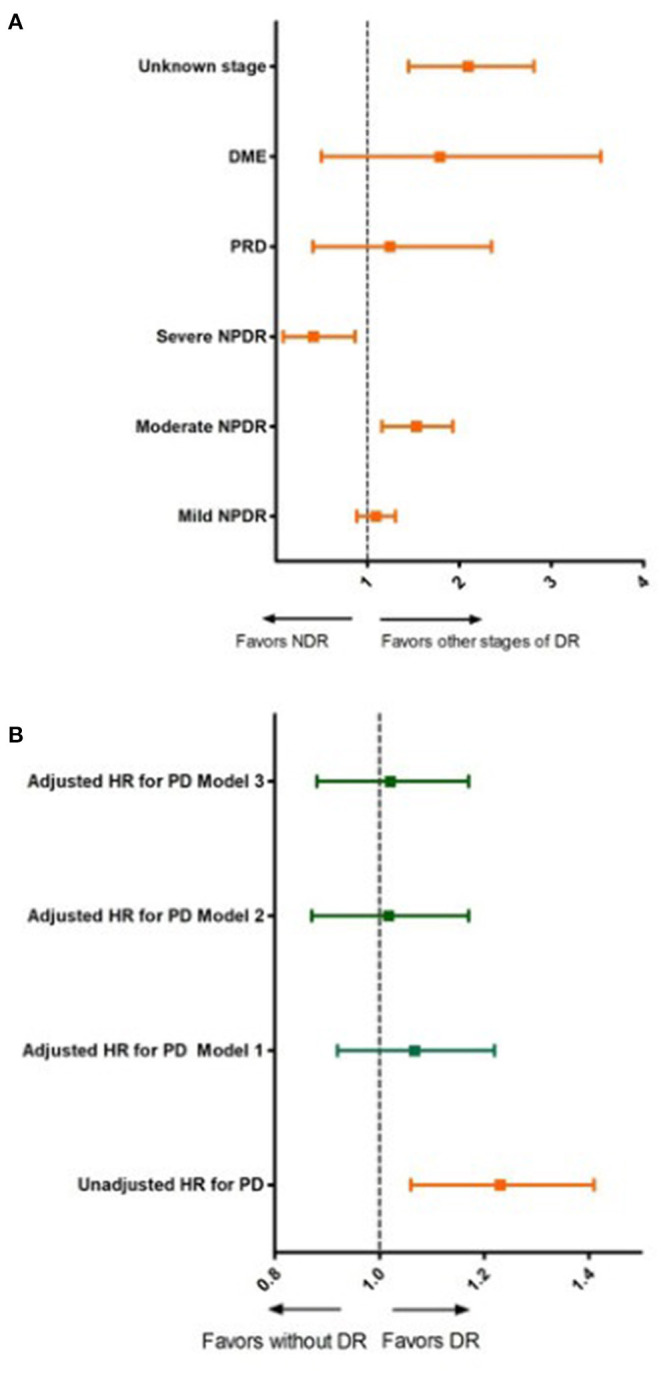
Unadjusted and adjusted hazard ratios for Parkinson's disease according to diabetic retinopathy status and stage of diabetic retinopathy. **(A)** Unadjusted hazard ratios for Parkinson's disease for different stages of diabetic retinopathy. DR, diabetic retinopathy; NDR, no apparent diabetic retinopathy; NPDR, non-proliferative diabetic retinopathy; PRD, proliferative diabetic retinopathy; DME, diabetic macular edema. **(B)** Unadjusted and adjusted hazards ratios for Parkinson's disease for subjects with and without diabetic retinopathy; Model 1: adjusted for age and sex; Model 2: adjusted for, age, sex, diabetes duration, smoking status, hypertension, dyslipidemia and body mass index; Model 3: adjusted for age, sex, diabetes duration, smoking status, hypertension, dyslipidemia, CKD, CVD and BMI and HbA1c.

Figure 2 and Table 4 show the results of the different multivariable proportional hazards analysis models. In the proportional hazards analysis adjusting the models for different risk factors, we did not find any statistically significant HRs for DR in any models. Age and male sex were independent risk factors in all of the models. T2DM duration was a risk factor in the second and third models, especially for subjects with T2DM duration of more than 20 years. In contrast, having a BMI over 39 kg/m^2^ and being an ex-smoker decreased the risk of PD. In the additional models, adjusting only for age, sex, and diabetes duration and/or HbA1c, we did not find a significant association of DR with PD. The age, sex and diabetes duration remained risk factors for PD in these models. Table 5 shows the results of these additional models.

**Table 4:** Adjusted hazard ratios for different variables.

	**Model 1**	**Model 2**	**Model 3**
Predictor	HR 95% CI [LI; UI]	HR 95% CI [LI; Ul]	HR 95% CI [LI; Ul]
Group with DR, ref: Group without DR	1.06 0.92; 1.22	1.01 0.87; 1.17	1.01 0.88; 1.17
Sex (male)	**1.35** **1.22; 1.48**	**1.07** **1.07; 1.08**	**1.38** **1.24; 1.54**
Age (years)	**1.08** **1.07; 1.08**	**1.39** **1.25; 1.55**	**1.07** **1.07; 1.08**
T2DM duration 6–10 years		**1.23** **1.10; 1.37**	**1.22** **1.10; 1.36**
T2DM duration 11–15 years		**1.24** **1.07; 1.43**	**1.23** **1.06; 1.43**
T2DM duration 16–20 years		**1.34** **1.07; 1.68**	**1.33** **1.06; 1.68**
T2DM duration more than 20 years		**1.57** **1.20; 2.05**	**1.56** **1.19; 2.04**
Ex-smoker		**0.69** **0.56; 0.84**	**0.69** **0.56; 0.84**
Current smoker		0.93 0.82; 1.05	0.92 0.82; 1.04
Dyslipidemia		1.07 0.97; 1.18	1.06 0.96; 1.17
Hypertension		0.94 0.85; 1.05	0.93 0.84; 1.04
BMI 24.9–29.9 kg/m^2^		1.02 0.86; 1.22	1.02 0.86; 1.22
BMI 30.0–34.9 kg/m^2^		0.94 0.78; 1.13	0.93 0.77; 1.12
BMI 35.0–39.9 kg/m^2^		0.90 0.70; 1.14	0.89 0.70; 1.14
BMI more than 39.9 kg/m^2^		**0.63** **0.41; 0.95**	**0.63** **0.41; 0.94**
BMI (missing)		0.93 0.77; 1.11	0.93 0.78;1.12
HbA1c 6.5–7%			0.98 0.85; 1.14
HbA1c 7.1–8%			1.00 0.87; 1.14
HbA1c 8.1–9%			1.08 0.90; 1.30
HbA1c 9.1–10%			0.96 0.73; 1.26
HbA1c more than 10%			**0.73** **0.53; 1.00**
HbA1c (missing)			0.93 0.81; 1.08
CKD			1.03 0.91; 1.17
CVD			1.08 0.95; 1.24
Observations	242,703	242,703	242,703
R2 Nagelkerke	0.026	0.027	0.027

*BMI, body mass index; CKD, chronic kidney disease; CVD, Cardiovascular disease; DR, diabetic retinopathy; HbA1c, glycosylate hemoglobin; HR, hazard ratio; 95% CI, 95% confidence interval; LI, lower limit; Ul, upper limit; T2DM, type 2 diabetes mellitus.*

*Bold values means statistically significant Hazard ratios*.

**Table 5:** Additional models for adjusted hazard ratios for different variables.

	**Model 1.1**	**Model 1.2**	**Model 1.3**
Predictor	HR 95% CI [LI; UI]	HR 95% CI [LI; Ul]	HR 95% CI [LI; Ul]
Group with DR, ref: Group without DR	1.01 0.88; 1.17	1.01 0.87; 1.16	1.06 0.92; 1.23
Sex (male)	**1.35** **1.23; 1.49**	**1.35** **1.23; 1.49**	**1.35** **1.23; 1.49**
Age (years)	**1.07** **1.07; 1.08**	**1.07** **1.07; 1.08**	**1.08** **1.07; 1.08**
T2DM duration 6–10 years	**1.23** **1.10; 1.38**	**1.23** **1.11; 1.38**	
T2DM duration 11–15 years	**1.25** **1.08; 1.44**	**1.36** **1.08; 1.70**	
T2DM duration 16–20 years	**1.36** **1.08; 1.70**	**1.25** **1.08; 1.44**	
T2DM duration more than 20 years	**1.59** **1.22; 2.08**	**1.59** **1.22; 2.08**	
HbA1c 6.5–7%	0.98 0.85; 1.13		1.00 0.87; 1.15
HbA1c 7.1–8%	0.99 0.87; 1.14		1.04 0.91; 1.19
HbA1c 8.1–9%	1.07 0.89; 1.29		1.14 0.95; 1.37
HbA1c 9.1–10%	0.95 0.72; 1.25		1.00 0.76; 1.32
HbA1c more than 10%	**0.72** **0.52; 0.99**		0.75 0.54; 1.03
HbA1c (missing)	0.92 0.80; 1.06		0.94 0.82; 1.08
Observations	242,703	242,703	242,703
R2 Nagelkerke	0.026	0.026	0.026

*HbA1c, glycosylate hemoglobin; HR, hazard ratio; 95% CI, 95% confidence interval; LI, lower limit; Ul, upper limit; T2DM, type 2 diabetes mellitus.*

*Bold values means statistically significant Hazard ratios*.

### Sensitivity Analysis

**Figure 3:**
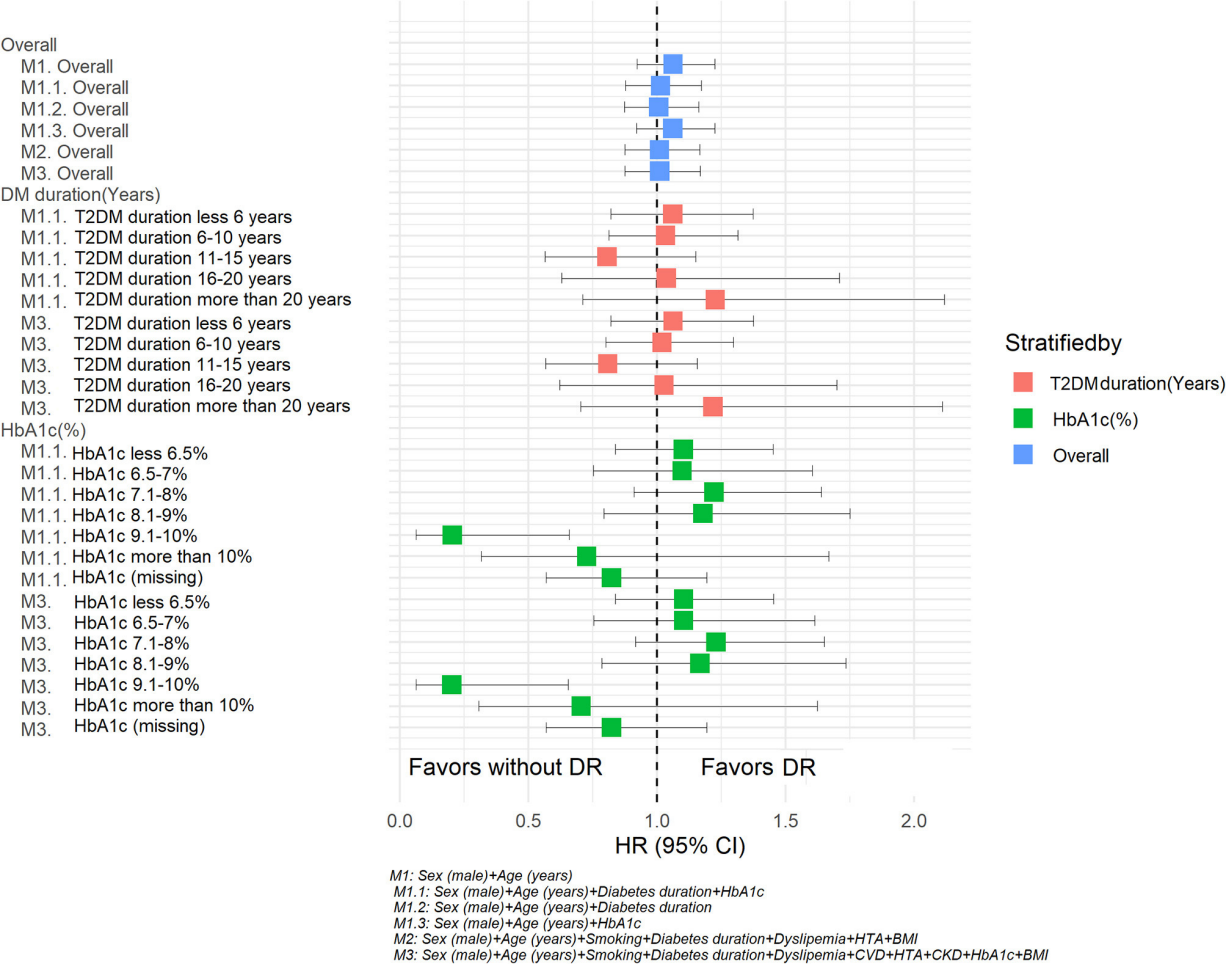
Hazard ratios for Parkinson's disease according retinopathy status. Sensitivity analysis. M1: Sex (male) + Age (years). M 1.1 Sex (male) + Age (years) + Diabetes duration + HbA1c. M1.2 Sex (male) + Age (years) + Diabetes duration. M1.3 Sex (male) + Age (years) + HbA1c. M2: Sex (male) + Age (years) + Smoking + Diabetes duration + Dyslipidemia + HTA + BMI. M3: Sex (male) + Age (years) + Smoking + Diabetes duration + Dyslipidemia + HTA +CKD + HbA1c + BMI.

In the sensitivity analysis, stratifying by diabetes duration or HbA1c, similar tendencies were observed for the HRs for DR observed in the previously described models. However, having an HbA1c between 9.1 and 10% and DR was negatively associated with PD when including additional relevant variables in this additional model (age, sex, T2DM duration) or variables in model 3 (age, sex, smoking, T2DM duration, dyslipidemia, CVD, HTA, CKD, IMC). The results of this sensitivity analysis are shown in Table 6 and Figure 3.

**Table 6:** Sensitivity analysis.

**Stratum**	**Subgroup**	**Model**	**HR**	**95% CI LI**	**95% CI Ul**	***p***	**Adjusted by**	**N**
Overall		1	1.06	0.92	1.23	0.40	Sex (male) + Age (years)	242,703
Overall		1.1	1.01	0.88	1.17	0.85	Sex (male) + Age (years) + T2DM duration + HbA1c	242,703
Overall		1.2	1.01	0.87	1.16	0.92	Sex (male) + Age (years) + T2DM duration	242,703
Overall		1.3	1.06	0.92	1.23	0.41	Sex (male) + Age (years) + HbA1c	242,703
Overall		2	1.01	0.86	1.17	0.89	Sex (male) + Age (years) + Smoking + T2DM duration + Dyslipidemia + HTA + BMI	242,703
Overall		3	1.01	0.88	1.17	0.88	Sex (male) + Age (years) + Smoking + T2DM duration + Dyslipidemia + CVD + HTA + CKD + HbA1c + BMI	242,703
T2DM duration	T2DM duration less 6 years	1.1	1.06	0.82	1.37	0.65	Sex (male) + Age (years) + HbA1c	138,553
T2DM duration	T2DM duration 6–10 years	1.1	1.03	0.81	1.31	0.79	Sex (male) + Age (years) + HbA1c	64,161
T2DM duration	T2DM duration 11–15 years	1.1	0.81	0.57	1.15	0.23	Sex (male) + Age (years) + HbA1c	27,449
T2DM duration	T2DM duration 16–20 years	1.1	1.04	0.63	1.71	0.89	Sex (male) + Age (years) + HbA1c	8,140
T2DM duration	T2DM duration more than 20 years	1.1	1.23	0.71	2.12	0.46	Sex (male) + Age (years) + HbA1c	4,400
HbA1c	HbA1c less 6.5%	1.1	1.10	0.84	1.45	0.49	Sex (male) + Age (years) + T2DM duration	79,068
HbA1c	HbA1c 6.5–7%	1.1	1.09	0.75	1.60	0.63	Sex (male) + Age (years) + T2DM duration	37,778
HbA1c	HbA1c 7.1–8%	1.1	1.22	0.91	1.64	0.18	Sex (male) + Age (years) + T2DM duration	41,252
HbA1c	HbA1c 8.1–9%	1.1	1.18	0.79	1.75	0.42	Sex (male) + Age (years) + T2DM duration	18,471
HbA1c	HbA1c 9.1–10%	1.1	0.21	0.06	0.66	0.01	Sex (male) + Age (years) + T2DM duration	9,607
HbA1c	HbA1c more than 10%	1.1	0.73	0.32	1.67	0.45	Sex (male) + Age (years) + T2DM duration	11,957
HbA1c	HbA1c (missing)	1.1	0.82	0.57	1.19	0.31	Sex (male) + Age (years) + T2DM duration	44,570
T2DM duration	T2DM duration less 6 years	3	1.06	0.82	1.38	0.64	Sex (male) + Age (years) + Smoking + Dyslipidemia + CVD + HTA + CKD + HbA1c + BMI	138,553
T2DM duration	T2DM duration 6–10 years	3	1.02	0.80	1.29	0.87	Sex (male) + Age (years) + Smoking + Dyslipidemia + CVD + HTA + CKD + HbA1c + BMI	64,161
T2DM duration	T2DM duration 11–15 years	3	0.80	0.57	1.16	0.25	Sex (male) + Age (years) + Smoking + Dyslipidemia + CVD + HTA + CKD + HbA1c + BMI	27,449
T2DM duration	T2DM duration 16–20 years	3	1.03	0.62	1.69	0.91	Sex (male) + Age (years) + Smoking + Dyslipidemia + CVD + HTA + CKD + HbA1c + BMI	8,140
T2DM duration	T2DM duration more than 20 years	3	1.22	0.70	2.11	0.48	Sex (male) + Age (years) + Smoking + Dyslipidemia + CVD + HTA + CKD + HbA1c + BMI	4,400
HbA1c	HbA1c less 6.5%	3	1.10	0.84	1.45	0.48	Sex (male) + Age (years) + Smoking + T2DM duration + Dyslipidemia + CVD + HTA + CKD + BMI	79,068
HbA1c	HbA1c 6.5–7%	3	1.10	0.75	1.61	0.61	Sex (male) + Age (years) + Smoking + T2DM duration + Dyslipidemia + CVD + HTA + CKD + BMI	37,778
HbA1c	HbA1c 7.1–8%	3	1.23	0.92	1.65	0.17	Sex (male) + Age (years) + Smoking + T2DM duration + Dyslipidemia + CVD + HTA + CKD + BMI	41,252
HbA1c	HbA1c 8.1–9%	3	1.17	0.78	1.73	0.45	Sex (male) + Age (years) + Smoking + T2DM duration + Dyslipidemia + CVD + HTA + CKD + BMI	18,471
HbA1c	HbA1c 9.1–10%	3	0.20	0.06	0.66	0.01	Sex (male) + Age (years) + Smoking + T2DM duration + Dyslipidemia + CVD + HTA + CKD + BMI	9,607
HbA1c	HbA1c more than 10%	3	0.71	0.31	1.62	0.41	Sex (male) + Age (years) + Smoking + T2DM duration + Dyslipidemia + CVD + HTA + CKD + BMI	11,957
HbA1c	HbA1c (missing)	3	0.82	0.57	1.19	0.31	Sex (male) + Age (years) + Smoking + T2DM duration + Dyslipidemia + CVD + HTA + CKD + BMI	44,570

*BMI, body mass index; CKD, chronic kidney disease; CVD, Cardiovascular disease; DR, diabetic retinopathy; HbA1c, glycosylate hemoglobin; HR, hazard ratio; 95% CI, 95% confidence interval; LI, lower limit; Ul, upper limit; T2DM, type 2 diabetes mellitus*.

## Discussion

The results from our retrospective cohort study from a primary care database from 2008 until 2016 showed no increased risk for developing PD among subjects with previous diabetic retinopathy in fully adjusted models. Instead, age, male gender, and longer T2DM duration conferred an increased risk of PD over time independently among T2DM subjects.

To date only one similar observational study with routinely collected health data from South Korea investigated the relationship between T2DM retinopathy and PD (16). Comparing the clinical characteristics of the DR subjects in both studies, subjects from our study were on average 6.7 years older, with a higher proportion of males (8.5%), 14.3% more smokers, and were also more obese (average difference 5.7 kg/m^2^). The differences found between subjects with DR from the two studies are not surprising. It is well reported that the characteristics of eastern Asian people with T2DM are different from European T2DM subjects (23). In Asian countries, T2DM subjects are younger and have a lower BMI than those from the US or European countries. Compared to Caucasians, Asians have more lipotoxicity and insulin resistance due to the greater visceral adiposity, which is more metabolically adverse (23). People with T2DM from Asia also tend to have a higher incidence of renal complications and ischemic strokes but lower coronary heart disease or peripheral arterial disease (24). Our study observed a higher percentage of subjects with CKD in the DR group (23.7%). However, these data cannot be compared to the South Korea study due differences in the definition of these variables. For instance, in the South Korean study, end-stage renal disease was defined by diagnostic code, while in our study, we defined CKD by values of CKD-EPI glomerular filtration rate <60 ml/min/1.73 m^2^ and/or an albumin/creatinine ratio >30 mg/g. Regarding the differences in the events of PD between the two studies, the incidence rate was slightly higher among subjects with DR in our study (16.95 vs. 15.51 per 10,000 person-years, respectively). Comparing groups without DR between the two studies, we observed a higher incidence rate than in the South Korea study (13.93 vs. 8.39 per 10,000 person-years, respectively). An explanation for this difference could be that our non-DR population was older than the South Korean population, and it is well known that PD increases rapidly with age (4). In general, our T2DM population was relatively older, and we had smaller differences between the groups in age (with or without DR). In particular, this could also explain the differences in the unadjusted HR observed in our study (Unadj-HR: 1.22 95% CI: 1.06; 1.41) compared with the South Korean study, where more pronounced differences in un-adjusted HR were observed (Unadj-HR: 5.72, 95% CI: 5.41; 6.05) (19). For our definition of DR, we intentionally included fundus photography, which is a gold standard screening method with a high sensitivity to detect DR, which could prevent possible misclassification of this condition (25). Possible overestimation of DR due to the utilization of only one diagnostic code related to DR (ICD-10: H36) in the South Korean study could be one of the reasons for significant associations found between DR and PD in the multivariable proportional hazards regression analysis models.

In our multivariable model, besides basic clinical factors such as age and sex, we evaluated the effect of duration of T2DM, BMI, smoking status and HbA1c. Longer diabetes duration has been previously established as a strong risk factor for microvascular complications, especially DR (26, 27). Indeed, we also observed that T2DM duration was an independent risk factor for the development of PD in our multivariable model analysis. Other similar studies have also reported that diabetes is an independent risk factor for PD. For example, a recently published observational study with the same database as ours reported an increased risk of PD among T2DM subjects (adj-HR of 1.19, 95% CI: 1.13; 1.25) and subjects with prediabetes (adj-HR of 1.07, 95% CI: 1.00; 1.14) compared to those subjects without these conditions (28). Despite the different periods of observation and definition of the study outcome, as well as the variable of exposure (prediabetes state and T2DM), a similar number of events for PD were observed in this study (PD among T2DM, 1,789 events) compared with our study (PD with and without DR, 1,748 events) (28). Knowing the fact that subjects with prediabetes are at an increased risk of developing T2DM (29), that the diagnosis of T2DM is usually delayed by between 4 and 7 years (30), and that both prediabetes and T2DM increase with age (31), could explain findings in our model that subjects with longer T2DM duration had a higher risk for PD. Similar results were also reported in a recently published case-control study from Denmark, in which the authors did not find an association between DR and PD; further, it should be pointed out that in this study subjects with Parkinson's disease had a longer duration of diabetes (32). In our sensitivity analysis, when we stratified for diabetes duration, the HRs remained in the same direction; however, there was no association of DR with the development of PD. Stratification for HbA1c revealed that a value between 9.1 and 10% in the presence of DR was negatively associated with PD. This finding has to be taken cautiously as it was only found for this HbA1c interval; additionally, there is no clear clinical explanation for this based on current knowledge.

The results from studies examining an association between BMI/obesity and PD have so far been inconsistent. For most of the longitudinal studies, according to a recent review on the epidemiology of PD, no associations between BMI and PD were observed (4). However, in a Spanish retrospective study, overweight and obese individuals were associated with higher PD risk (adj-HR: 1.21; 95% CI 1.15–1.27), compared with normal weight (28). This could be explained due to the different population characteristics or different variables considered in the HR analysis. Another prospective study from Finland reported that overweight or obese subjects were more at risk of developing PD than those with a BMI <23 kg/m^2^ (33). We observed surprising results in our multivariable models, where those subjects with a BMI >39.9 kg/m^2^ had a 41% decreased risk of developing PD compared with subjects with a BMI <24.9 kg/m^2^. We do not have an explanation for this association which was otherwise only restricted to the highest stratum of body weight. Therefore, further studies are warranted to address this question.

So far one recent systemic review and meta-analysis, including 27 observational studies, addressed the relationship between DR and systemic neurodegeneration (34). The authors reported that systemic neurodegeneration was statistically associated with DR. However, in a smaller subgroup meta-analysis related to the severity of DR, the authors did not observe associations with systemic neurodegeneration. Moreover, they only included one study that analyzed the relationship between DR and PD that was summarized but not included in the quantitative review (34). Another systematic review on the potential association between diabetic retinopathy and hyperkinetic disorders found that DR could be indirectly related with striatopallidal microangiopathy; further, the authors suggested that the severity of the DR and glycemic fluctuation could be associated with increased risk or worse prognosis of hyperkinetic disorders (35).

The results of our study should be interpreted with caution, keeping in mind potential limitations. Firstly, this was a retrospective observational study with pseudo-anonymized routinely collected health data where the presence of missing data is inherent. There is a possibility that not all subjects with DR were captured. For this reason, we used the fundus photography results and combined them with diagnostic codes. Secondly, a numerical imbalance between the different groups occurred due to the study design and lack of randomization. Thirdly, we did not have information on the treatment of PD; therefore, we only used the diagnostic code to define the main study event. Besides these limitations, our study includes a large population of subjects attended in primary health care centers which increases the statistical power. Moreover, in our multivariable hazard ratio models, we considered more potentially confounding variables (T2DM duration) related to PD than previously published studies.

In conclusion, in our primary health care population database, DR was not associated with an increased risk of PD after adjusting for different risk factors. The detection of DR is mainly based on retinal vascular changes that do not always coincide with retinal neurodegeneration (33). Even though a strong biological background exists between DR and PD, further mechanistic studies and well-designed clinical studies are needed to investigate the possible relationship between these two neurodegenerative conditions.

## Data Availability Statement

The data analyzed in this study is subject to the following licenses/restrictions: restrictions apply to the availability of some or all data generated or analyzed during this study because they were used under license. The corresponding author will on request detail the restrictions and any conditions under which access to some data may be provided. Requests to access these datasets should be directed to Dídac Mauricio, didacmauricio@gmail.com.

## Ethics Statement

The studies involving human participants were reviewed and approved by IDIAP Jordi Gol i Gurina Foundation (protocol code P13/028 and date of approval 03/04/2013). Written informed consent for participation was not required for this study in accordance with the national legislation and the institutional requirements.

## Author Contributions

DM: conceptualization and methodology. JR: software, formal analysis, and data curation. JR, BV, and DM: validation. XM-T: resources. BV: writing—original draft preparation. BV and DM: writing—review and editing. DM, JJ, JR, EO, EC, JB, JK, and JF-N: supervision. All authors have read and agreed to the published version of the manuscript.

## Conflict of Interest

DM and JF-N received advisory and/or speaking fees from Astra-Zeneca. Ascensia. Boehringer Ingelheim. GSK. Lilly. MSD. Novartis. Novo Nordisk. and Sanofi and received research grants to the institution from Astra-Zeneca. GSK. Lilly. MSD. Novartis. Novo Nordisk. Sanofi. and Boehringer. EO has received advisory and or speaking fees from Astra-Zeneca, Boehringer Ingelheim, Lilly, MSD, Novo Nordisk, Sanofi, and Amgen; he has received research grants to the institution from MSD and Amgen. The remaining authors declare that the research was conducted in the absence of any commercial or financial relationships that could be construed as a potential conflict of interest.

## Publisher's Note

All claims expressed in this article are solely those of the authors and do not necessarily represent those of their affiliated organizations, or those of the publisher, the editors and the reviewers. Any product that may be evaluated in this article, or claim that may be made by its manufacturer, is not guaranteed or endorsed by the publisher.
